# Supplementary material for: Clinical Trypanosoma cruzi isolates share a common antigen repertoire that is absent from culture adapted strains
Source: bioRxiv. 2025 Jun 4:2025.06.04.657671. Preprint. [Version 1] doi: 10.1101/2025.06.04.657671 (PMC12157461; doi:10.1101/2025.06.04.657671)
Supplement: Supplement 3 [file media-3.pdf]

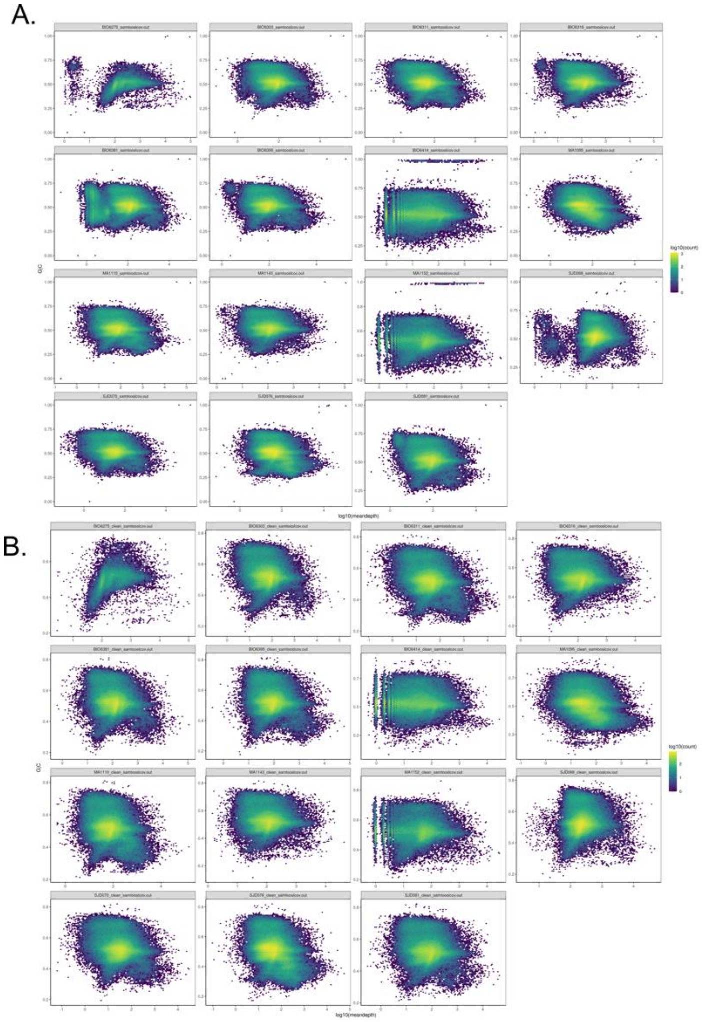

Supplemental Figure 1: 2D histograms of GC percent and contig coverage for clinical isolate assemblies before (A) and after (B) cleaning contaminating DNA and kDNA

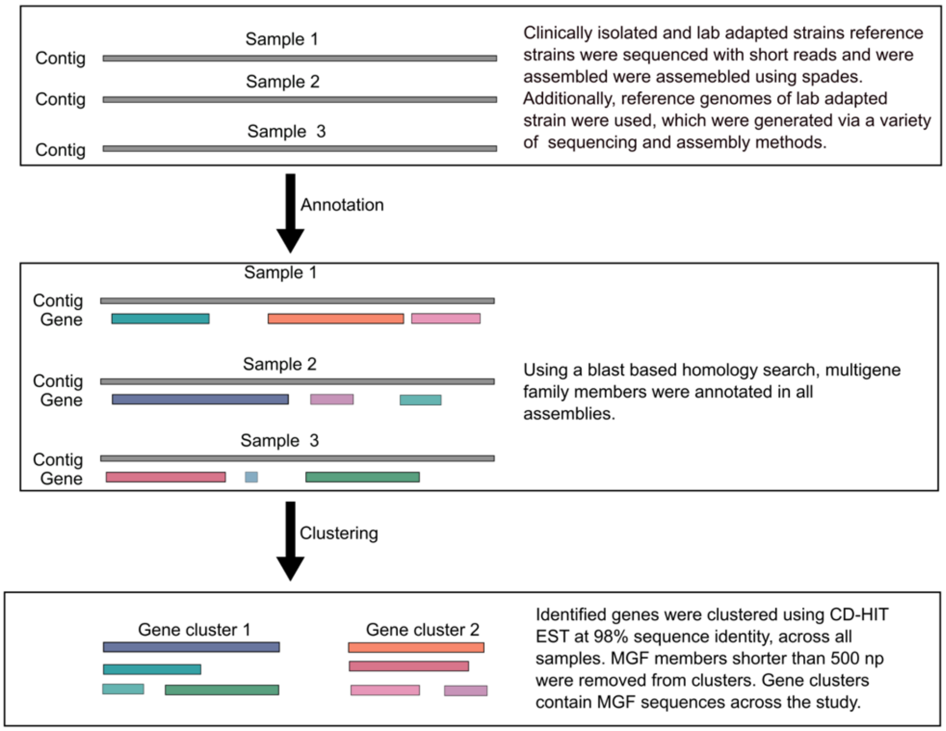

Supplemental Figure 2: Schematic representation of gene clustering strategy for multigene family members

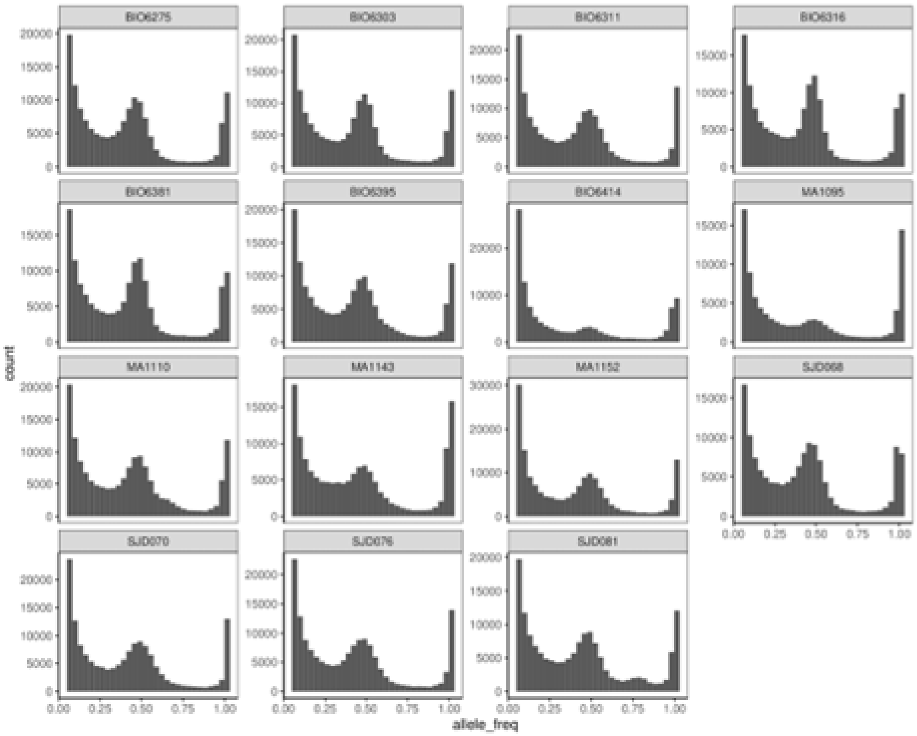

Supplemental Figure 3: Distribution of allele frequency for clinically isolated strains against the CL Brener Esmeraldo-like reference genome. Isolates BIO6414 and MA1095 have smaller peaks at 0.5, due to their non-hybrid nature, and lower prevalence of heterozygous alleles.

Sample ID	numreads_R1	numreads_R2	HE	Haploid Genome length	Assembly len	# Contigs	Average Contig Length	n50	Single (%)	Duplicated (%)	Fragmented (%)	Incomplete (%)	Missing (%)
BIO6275	3463629	3512846	2.49455	40818222	46568153	28140	1654	3943	77.69	7.69	13.08	0.77	0.77
BIO6303	2452701	2432325	2.34576	37324623	69848654	126469	552	1813	51.53	16.92	27.69	0.77	3.08
BIO6311	1615176	1582604	2.48213	40271855	72304979	151300	477	1512	62.30	15.38	20.77	0.00	1.54
BIO6316	3971510	3949092	2.41809	38018721	70828901	128911	549	1862	44.61	16.92	34.62	0.77	3.08
BIO6381	4095168	4101251	2.36717	37091970	71230598	131032	543	1763	46.92	16.15	33.08	0.00	3.85
BIO6395	2961480	3002744	2.51745	40035111	71052600	135798	523	1780	57.69	16.15	23.85	0.77	1.54
BIO6414	1793955	1774465	1.0475	37618289	57681790	156709	368	770	100	0.00	0.00	0.00	0.00
MA1095	1804518	1798788	0.984298	42523145	58393558	174822	334	627	100	0.00	0.00	0.00	0.00
MA1110	2825007	2820456	2.44574	40467366	73701203	153886	478	1561	48.46	15.38	31.54	0.77	3.85
MA1143	3421261	3419717	2.48146	39985326	69584163	122999	565	2003	44.61	17.69	33.85	0.77	3.08
MA1152	1439860	1451530	2.52186	39394166	62347121	104101	598	2061	63.07692	10.77	24.62	0.00	1.54
SJD068	6413595	6601890	2.54625	38631308	70882885	137934	513	1523	55.38462	11.54	29.23	1.54	2.31
SJD070	1187680	1168239	2.58895	39728401	67533274	136496	494	1527	73.07692	8.46	16.92	0.00	1.54
SJD076	2000918	2022794	2.48158	44142051	79778165	196128	406	1244	57.69231	11.54	27.69	0.77	2.31
SJD081	2743003	2707380	2.47528	39546644	70022023	127496	549	1854	49.23077	16.15	30.77	0.77	3.08

Supplemental Table 1: Genome assembly statistics.

Population	Feature	Aggregated metric
Peripartum (N=4)	Mean Age	28.20
	% Men	0%
	Parity (mean count)	2.75
	Known vector exposure (% with known exposure)	25%
	Transmitted to infant	25%

	(% Transmitted)	
Persons with HIV (N=4)	Mean Age	47.75
	% Men	100%
	CD4 Count (mean count)	278.3
	HIV Load (mean copies/ml)	33,364.66
	Micromethod Result (% positive)	75%
	Mean *T. cruzi* parasite load (parasites/ml)	1,449,762.25
Participants in study of Chagas cardiomyopathy (N=6)	Mean Age	56
	% Men	75%
	% with ECG abnormalities (count)	42.8% (3)

Supplemental Table 2: aggregated characteristics of study population

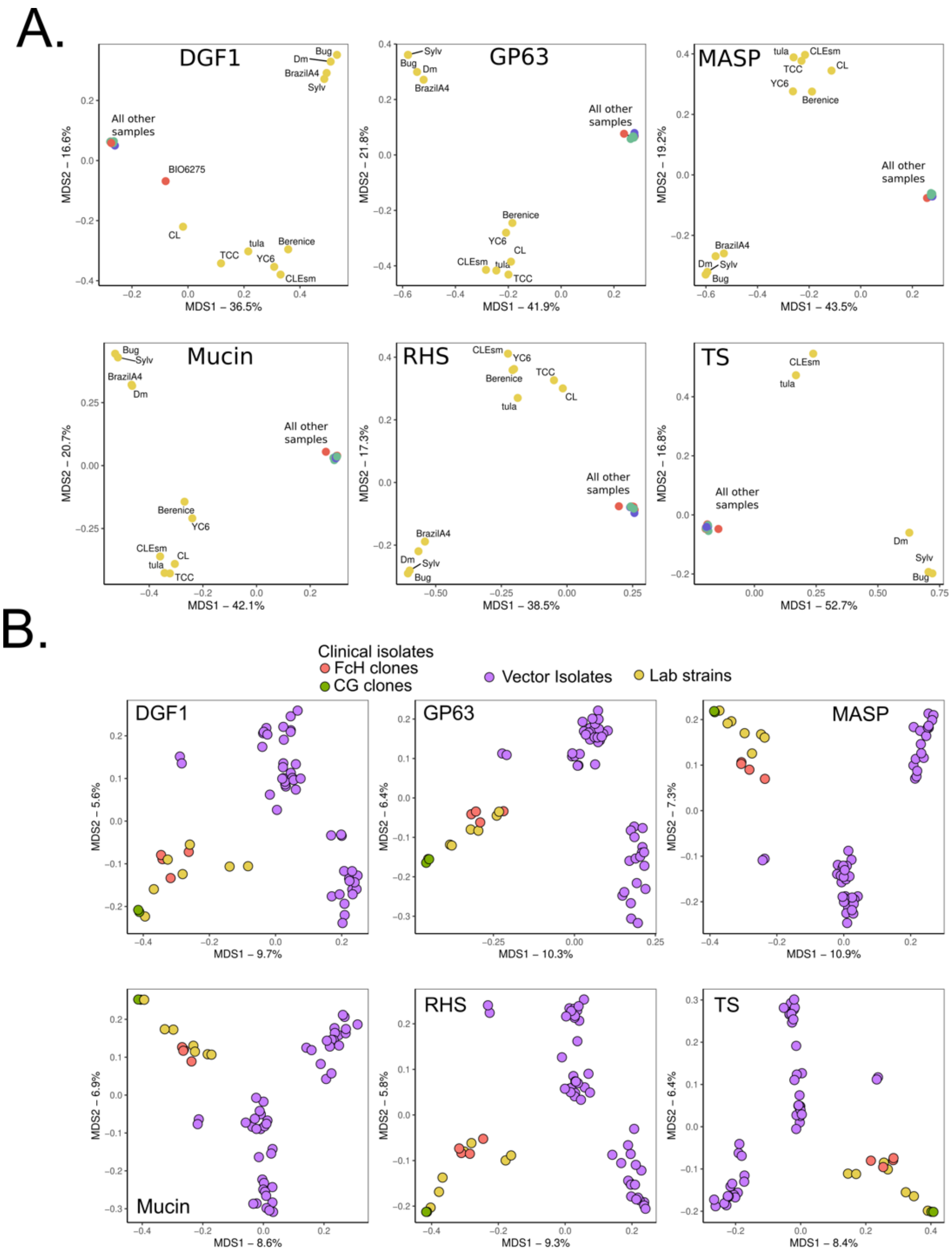

Supplemental Figure 4: A) MGF repertoire separation including Tc1 lab reference strains. DTU separation is more pronounced but note that due to the lack of clinically isolated specimens in the current study some separation may be largely DTU driven. B) MGF repertoire separation of TcI parasites alone, including Colombian clinically isolated parasites from Talavera-Lopez et al (FcH and CG, red and green respectively) and vector isolates from Schwabl et al. (purple). FcH and CG are two individuals for which there were four and two parasite genomes cloned from the patient samples

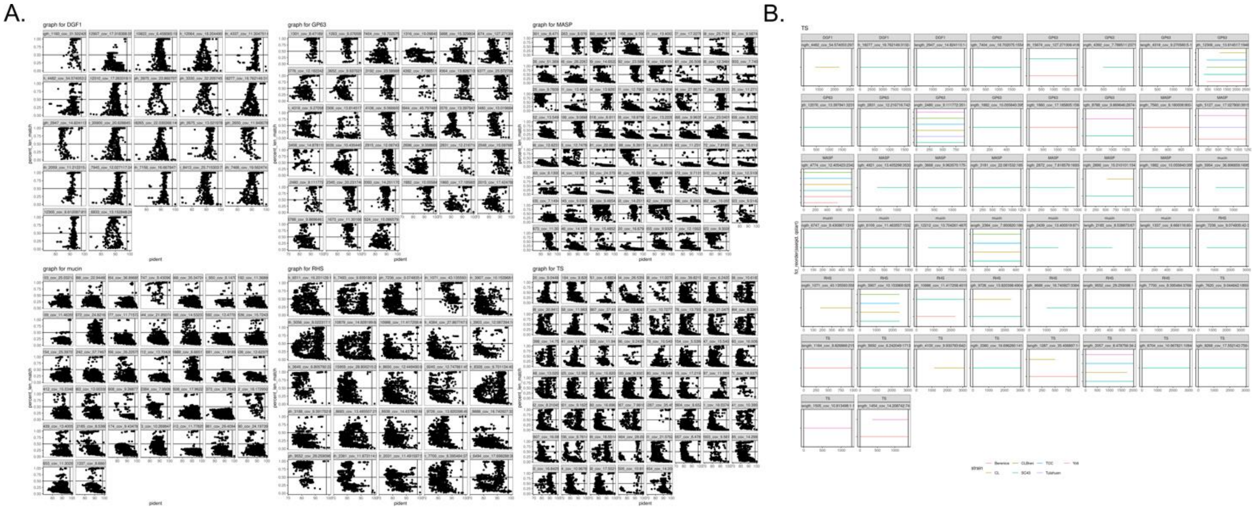

Supplemental Figure 3: Confirming the loss of shared MGFs among lab adapted strains by BLASTN search of lost representative sequences within whole genomes of TcII, TcV and TcVI strains. **A)** Plot of all BLASTN hits in reference genomes for each lost MGF encoding gene, across all gene families. X axis represents the percent identity of the BLASTN hit, and Y axis represents the percent of the total MGF that is covered by the BLASTN homology found in the reference genome. The horizontal line is at 50% query coverage, and the vertical line is at 98% BLASTN sequence identity. **B)** Each MGF member that passes the above thresholds. Each gene is a facet, colored by the reference genome the gene was found in. X axis is the length of the gene, with black bars demarcating that start and end, and line segments of each reference genome representing the position where the hit occurred. These are the genes that were removed from the set of shared clinical MGF members that were lost in the lab adapted strains.
